# Optimizing the Surface Functionalization of Peptide–MXene Nanoplatforms to Amplify Tumor-Targeting Efficiency and Photothermal Therapy

**DOI:** 10.34133/bmr.0198

**Published:** 2025-05-26

**Authors:** Sujin Kim, Sathiyamoorthy Padmanaban, Aravindkumar Sundaram, Gul Karima, In-Kyu Park, Hwan D. Kim

**Affiliations:** ^1^ Department of Polymer Science and Engineering, Korea National University of Transportation, Chungju 27469, Republic of Korea.; ^2^ Department of Biomedical Sciences and BioMedical Sciences Graduate Program (BMSGP), Chonnam National University Medical School, Hwasun 58128, Republic of Korea.; ^3^ DR.Cure, Hwasun 58128, Republic of Korea.; ^4^ Department of Biomedical Engineering, Korea National University of Transportation, Chungju 27469, Republic of Korea.

## Abstract

Energy storage and conversion extensively use MXenes, a class of 2-dimensional transition metals. Research is currently exploring MXenes in areas such as biomedical imaging, positioning them as a substantial contender in biomedical applications. Even though these biocompatible MXenes have many uses, it is challenging to make nanoparticles that are all the same size. This has made it harder to use them in the biomedical field. Herein, we meticulously crafted nano-sized MXene particles, achieving exceptional uniformity and amplified photothermal conversion efficiency compared to those of their bulkier micro-sized counterparts. To make these nanoparticles better at finding tumors, we added ARGD peptides to their surfaces. These are biomolecules that are known to bind to integrin α_v_β_3_, a protein that is highly expressed in cancerous cells. Our research showed that these RGD–MXene nanoconjugates have excellent targeting accuracy and can eradicate tumors very effectively. This targeted photothermal therapy platform promises to redefine cancer treatment by selectively eradicating malignant cells while safeguarding healthy tissue. Also, MXene’s natural ability to change surfaces opens up a world of possibilities for a wide range of uses in nanomedicine, bringing about a new era of sophisticated therapeutic interventions.

## Introduction

Cancer is a debilitating disease and a significant contributor to mortality among noncommunicable diseases [1]. In recent years, there has been a notable increase in cancer-related deaths, underscoring the urgent need for a tumor treatment modality that offers high efficacy with minimal side effects [2]. Conventional tumor treatment modalities include surgical resection, chemotherapy, and radiation therapy [3]. However, these approaches are associated with potential risks from high-energy radiation and the side effects of pharmacological agents [4]. Photothermal treatment has emerged as a promising approach to addressing these limitations. Photothermal therapy (PTT) is designed to eradicate tumor cells by harnessing the temperature elevation resulting from the conversion of light energy into heat. This technique offers the advantages of straightforward procedures, abbreviated treatment duration, and rapid recovery and represents an effective noninvasive treatment modality capable of targeting various types of tumors [5]. The PTT process relies on the use of photothermal agents, which convert light into heat [6]. These agents accumulate within the tumor and are irradiated with a near-infrared (NIR) laser to induce temperature elevation. Notably, tumor cells are susceptible to destruction at temperatures exceeding 45 °C, thereby facilitating tumor eradication [7]. This localized heat transfer approach is being investigated as an efficient tumor ablation method, reducing the need for extensive equipment compared to alternative treatments. Nanoparticles are promising photothermal agents due to their potential for enhanced therapeutic effects [8,9]. As materials transition in size from the macroscale to the nanoscale, they exhibit altered optical, magnetic, and electrical properties, influencing their behavior [10]. Nanoparticles demonstrate enhanced photothermal conversion efficiency due to their large surface-area-to-volume ratio. Furthermore, the compromised endothelium of blood vessels surrounding tumor cells presents gaps within the vascular barrier, allowing nanoparticle accumulation within tumor sites. Consequently, the integration of nanotechnology into tumor treatment has the potential to mitigate the drawbacks associated with chemotherapy.

Materials like gold and graphene are commonly used as photothermal agents due to their excellent thermal conductivity and metallic properties [11,12]. The discovery of graphene in 2004 sparked a growing interest in the exploration of 2-dimensional (2D) nanomaterials [13,14]. Among metallic materials, MXene, a 2D nanomaterial with the chemical formula M*_n_*X_*n*+1_, where M is a transition metal and X is a nitride or carbide, was discovered in 2011 and has been actively studied since then [15]. Various combinations of MXenes can be produced through the combination of transition metals such as Nb and Mo [16]. Particularly, the recently developed Ti_3_C_2_ MXene exhibits exceptional photothermal properties and conductivity [17]. MXene has been found to exhibit biocompatibility within a specific concentration range [18,19]. The biocompatibility and toxicity of MXene are dependent on concentration. Dmytriv and Lushchak [20] summarized the toxicity of MXene to cell cultures. For biological studies, it is important to select the dose of an apparent substance that does not induce toxicity. It has been shown that no toxicity is observed at low concentrations below about 1,000 μg/ml. Additionally, the surface of MXene contains functional groups such as –O, –F, and –OH, making it applicable to various fields through facile modification [21]. However, when MXene is applied to PTT, it may affect normal cells as it lacks the ability to target tumors selectively. High concentrations of MXene are required to achieve effectiveness in the treatment process, leading to increased cytotoxicity and concerns about potential side effects. To address this issue, MXene needs to be modified to enable specific tumor cell targeting.

Achieving specific tumor targeting requires endowing the materials with specific functions. While the primary method of passive delivery involves nanoparticle accumulation at tumor sites due to the enhanced permeability and retention (EPR) effect [22], this approach may only sometimes be adequate for specific targeting [23]. To overcome this limitation, a strategy targeting integrin α_v_β_3_, which is overexpressed in tumor cells, has been established. Integrin α_v_β_3_ plays a crucial role in tumor development, epithelial–mesenchymal metastasis, vascular development, and drug resistance [24]. RGD peptides, which bind to integrin α_v_β_3_, offer a promising avenue for selectively targeting tumor cells. RGD peptides can bind to integrin receptors on the cell surface, and certain integrins identify RGD motifs in proteins and bind to these peptides, enabling cell adhesion and communication [25]. In general, most integrins have active and inactive states. The extracellular domain of integrin α_v_β_3_ is bent or folded in the inactive state to prevent binding, whereas in the on state to which the RGD peptide is bound, the extracellular domain appears straight without being bent. The mechanism by which the RGD peptide binds to integrin α_v_β_3_ has been shown to form a double-bond salt bridge by replacing an amine in the guanidine group of the ligand [26]. In addition, RGD peptides targeting integrin α_v_β_3_ have cancer target characteristics and play a role in accurately delivering drugs in treatment. In addition, since cancer cells are targeted specifically, damage to healthy cells can be minimized [27]. However, RGD peptides are derived from natural proteins and are easily degraded by proteases in the body. To overcome this problem, a modified or cyclic RGD peptide is used [28]. Also, the cyclic RGD peptide binds more strongly to integrin v3 compared to the linear RGD. The application of such an RGD peptide may actively target tumors and may be a strategy for efficiently delivering a tumor therapeutic agent in cancer treatment.

**Fig. 1.**
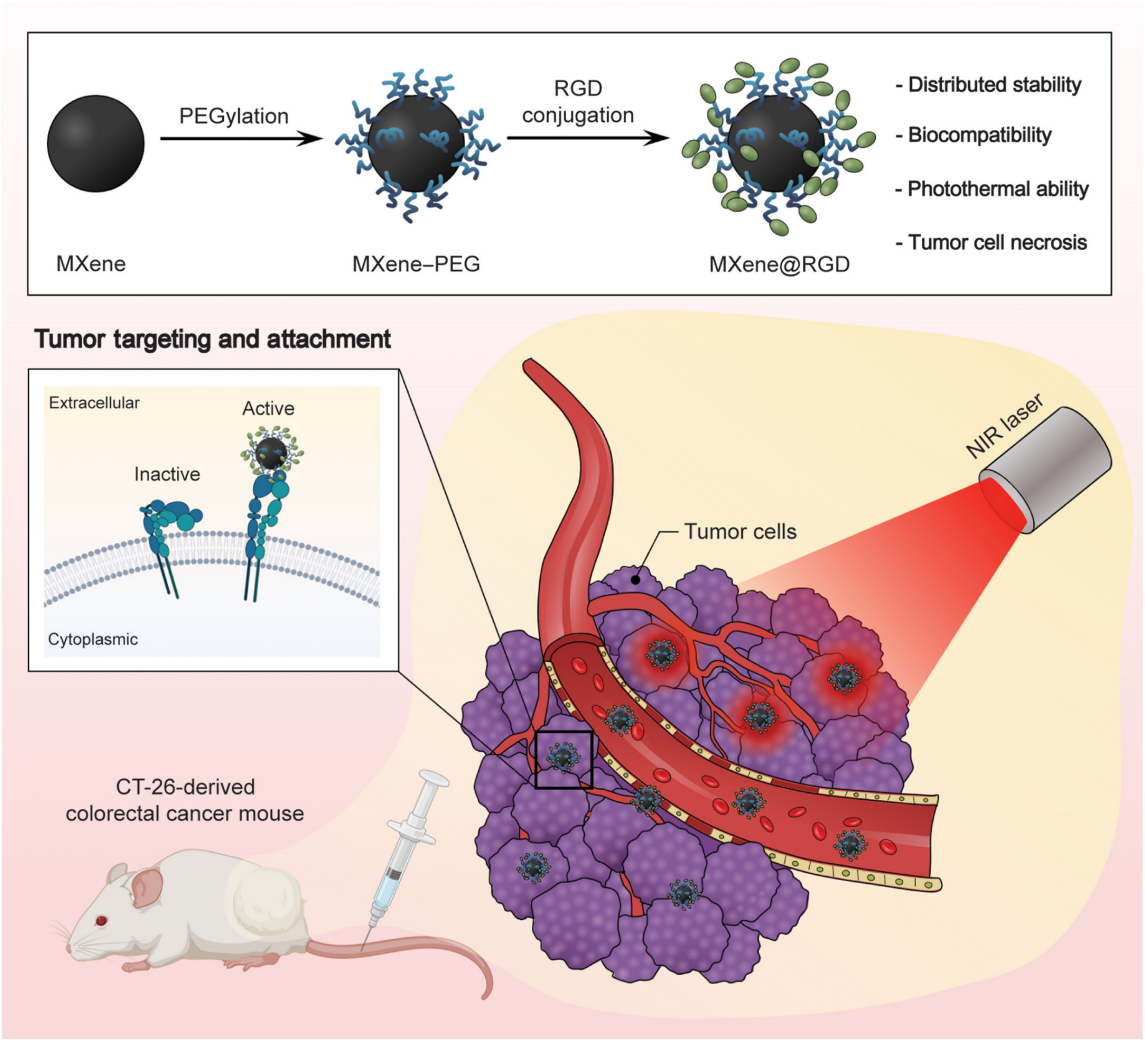
Schematic diagram of a Ti_3_C_2_ MXene-based tumor-targeting system for in vivo synergistic photothermal therapy of tumor, including transport within the blood vessel and accumulation into a tumor. NIR, near-infrared.

This study aimed to investigate the tumor treatment potential of modified MXene with a targeting function that binds to integrin α_v_β_3_, overexpressed in tumor cells [29]. Prior to the modification of MXene, the photothermal effect of the material was demonstrated. The adjustment of MXene to micro and nano sizes was conducted to assess the photothermal conversion and tumor cell death characteristics based on size. Subsequently, the MXene nanoparticles were modified with RGD peptides, and their properties were analyzed (MXene@RGD). The tumor-targeting ability of MXene, both before and after modification, was evaluated using tumor cells and normal cells. This targeted approach has the advantage of minimizing the impact on surrounding normal cells and specifically targeting tumor cells, thereby reducing the amount of MXene required. Moreover, in experiments with HeLa, a cervical cancer cell, and CT-26, a colorectal cancer cell, the applicability of MXene@RGD to various tumors was suggested. The ultimate goal is to establish a tumor treatment system capable of targeting tumor cells and inducing therapeutic effects through photothermal conversion (Fig. 1).

## Materials and Methods

### Materials

Ti_3_C_2_ MXene flakes were procured from Haydale Technologies Co., Ltd. (MXNTI3C2TX-FLN, UK). 1,4-Dioxane (072622) and ethyl ether (112822) were obtained from Samchun, Republic of Korea. (3-Aminopropyl)triethoxysilane (440140, USA) was sourced from a supplier in the USA, while poly(ethylene glycol) (PEG) was obtained from a supplier in Switzerland. Succinic anhydride was sourced from a supplier in Italy, and 4-(dimethylamino)pyridine (107700, USA), triethylamine (471283, USA), and *N*-hydroxysuccinimide (130672, Japan) were obtained from Sigma-Aldrich. 1-(3-Dimethylaminopropyl)-3-ethylcarbodiimide (EDC) was procured from a supplier in Japan, and Cyclo (–RGDyK, AS-61183, USA) peptide was obtained from AnaSpec, USA.

### Size control of MXene particles

MXene flakes were prepared in distilled water at a concentration of 1 mg/ml and dispersed at an ultrasonic frequency of 40 kHz for 1 h. In this process, a solution of microparticles was obtained. Part of this solution was centrifuged at 3,500 rpm and 4 °C for 1 h to bring down large particles. The supernatant of the solution was extracted to obtain a solution of nanoparticles. The microparticles and nanoparticles obtained through this process were measured in size using dynamic light scattering (DLS; ELS-Z2, Otsuka Electronics Co., Ltd., Japan), which measures the speed of Brownian motion, which varies depending on the large size of the particles.

### Cell culture

HeLa cells were obtained from Incheon National University, and mesenchymal stem cells (MSCs) were obtained from Seoul National University. In addition, CT-26 was purchased from the Korean Cell Line Bank (74364). HeLa and CT-26 were cultured in Dulbecco’s modified Eagle medium (#1000136026, STEMCELL, Canada) supplemented with 10% fetal bovine serum (26140079, Gibco, USA) and 1% penicillin–streptomycin–glutamine (10378016, Gibco, USA). MSCs were cultured in MesenCult Proliferation Kit (Human) (ST05411, STEMCELL, Canada) supplemented with 10% fetal bovine serum and 1% penicillin–streptomycin–glutamine. All cells were cultured in a 5% CO_2_/95% O_2_ incubator at 37 °C, supplemented with fresh medium every 2 to 3 d.

### Fabrication of tumor spheroids according to MXene particles

To simulate 3-dimensional-type tumors, tumor spheroids were manufactured using a platform called LabSphero (2035080, LabToLab, Republic of Korea). Prior to seeding the cells, LabSphero was washed 3 times with ethanol (EtOH; 000E0219, Samchun, Republic of Korea) and once with phosphate-buffered saline (PBS) (ST37350, STEMCELL, Canada). Moreover, HeLa cells were seeded at 1 × 10^6^ cells/dish cell density. Cells were cultured within the platform for 2 d, formed spheroids, and obtained on the last day. The diameter and aspect ratio of the spheroids were quantified through the ImageJ software (National Institutes of Health, USA).

### Photothermal performance of MXene particles

To evaluate the photothermal performance of MXene particles, the control group without MXene particles, the group with MXene microparticles (10 and 50 μg/ml), and the group with nanoparticles (10 and 50 μg/ml) were irradiated with NIR laser (MDL-III-808, CNI Laser, China) at a power of 1.5 W/cm^2^ for 10 min. In addition, different 808-nm laser power densities (0.5, 1.0, and 1.5 W/cm^2^) were irradiated to each group for 10 min. Alternatively, each group was treated with 5 cycles, 3 min each, for heating and cooling to 1.5 W/cm^2^. The temperature was measured with a thermal imaging camera (E5, FLIR, Estonia). Subsequently, laser-irradiated tumor spheroids were stained using the LIVE/DEAD assay (L3224, Invitrogen, USA) to assess the tumor-killing ability of MXene particles. Images were captured and visualized using a fluorescence microscope with the ZEN lite software. Cell viability was calculated as the number of living cells relative to the total number of cells. In addition, the photothermal conversion efficiency (*η*) of MXene was calculated using the formula below:

$$\eta =\frac{\mathrm{hA}\left(T_{\max}-T_{\mathrm{amb}}\right)-Q_{\text{loss}}}{I\left(1-10^{-A_{\lambda }}\right)}.$$ (1)

Here, *h* is the convective heat transfer coefficient, *A* is the surface area of the sample, *T_max_* is the maximum temperature, *T_amb_* is the ambient temperature, *I* is the incident light intensity, and *A_λ_* is the absorbance.

### Carboxylation of PEG

PEG was carboxylated to modify the surface of MXene particles; 5.00 g of PEG and 0.25 g of succinic anhydride were dissolved in 10 ml of 1,4-dioxane, and an additional 122 mg of 4-(dimethylamino)pyridine and 0.25 ml of triethylamine were added and stirred at room temperature for 24 h. Then, the mixed solution was precipitated with 15 ml of cold ethyl ether and centrifuged at 1,100 rpm for 5 min to remove the supernatant, a monomer that did not participate in the reaction. A portion of the remaining solution was dispersed in 10 ml of distilled water, and PEG–COOH powder was obtained using a freeze dryer (Tabletop Freeze Dryer, TFD8501, ilShinBioBase, Republic of Korea).

### PEGylation and RGD peptide conjugation of the MXene surface

First, 30 μl of (3-aminopropyl)triethoxysilane was added to 3 ml of MXene solution and stirred at room temperature for 12 h to create amino-functionalized MXene capable of binding to PEG–COOH. Then, 12 μl of EDC and 2.58 μl of *N*-hydroxysuccinimide were added and stirred for 30 min to activate amino groups, followed by the addition of 30 mg of PEG–COOH and stirring at room temperature for 24 h. Afterward, 1.8591 mg of RGD peptide was added and stirred at room temperature for another 24 h. The solution was then centrifuged at 15,000 rpm for 30 min, and the supernatant was discarded to remove unreacted monomers. After washing 3 times, the final product, MXene@RGD, was dispersed in deionized water (DW).

### Characterization of MXene@RGD

To confirm the synthesis of MXene@RGD, Fourier transform infrared (FTIR) spectroscopy (FT/IR-4600LE, JASCO, Japan) with attenuated total reflectance (ATR) accessories was used to record spectra from 400 to 4,000 cm^−1^. In addition, DLS was used to measure particle size. A comparison was made between MXene before and after synthesis, and stability was evaluated by dispersing the samples in different solvents. Unmodified MXene and MXene@RGD were dispersed in DW, EtOH, and dimethyl sulfoxide (D8418, Sigma-Aldrich, USA), respectively. They were observed again after being left at a dark room temperature for 7 d. In addition, the zeta potential was measured to show the change in the surface charge of MXene@RGD before and after synthesis (ELSZneo, Otsuka Electronics Korea Co., Ltd., Japan).

### Immunofluorescence staining of spheroids

HeLa cells, CT-26, and MSCs were made into spheroid form by LabSphero. Spheroids of each of the 3 cell types were obtained on day 2 of LabSphero culture and attached to gelatin-coated coverslips for microscopic observation. The spheroid was fixed using 3.6% paraformaldehyde (47608, Sigma-Aldrich, Switzerland) for 10 min and then washed with PBS. To increase permeability, it was incubated with 0.5% Triton X-100 (T8787, Sigma-Aldrich, USA) solution for 15 min and then washed. Then, it was incubated at room temperature for 1 h in a solution where 0.2% bovine serum albumin (A7906, Sigma-Aldrich, USA) and 0.1% Triton X-100 solution were mixed in a 1:1 ratio. After being washed, the primary antibody was incubated overnight at 4 °C in a solution mixed in PBS at a ratio of 1:500. After washing 3 times with 0.1% Triton X-100 solution, it was cultured for 1 h in the dark at room temperature using a secondary antibody. After washing 3 times, it was incubated for 5 min with 4′,6-diamidino-2-phenylindole (DAPI) (D1306, Invitrogen, USA) in the dark at room temperature and then washed and observed for spheroids through a fluorescence microscope. The fluorescence expression regions of the spheroids were quantified using the ImageJ software (National Institutes of Health, USA). To measure the fluorescence expression intensity, HeLa, CT-26, and MSCs were seeded at 50,000 cells/well in 96-well plates. When cells grew and filled the bottom, they were fixed using 3.6% paraformaldehyde, and immunofluorescence staining was performed using the same method as earlier. Each fluorescence intensity was measured using a microplate reader (Infinite 200 Pro, Tecan, Swiss). Fluorescence intensity measurements used a red wavelength band (excitation: 590 nm; emission: 617 nm) and a green wavelength band (excitation: 488 nm; emission: 496 nm). Of the primary antibody used for immunofluorescence staining, integrin α_v_β_3_ (bs-1310R, polyclonal immunoglobulin G) was purchased from Bioss Antibodies, and Ki-67 (14-5699-82, monoclonal) and annexin V (YB3832734A, polyclonal) were purchased from Invitrogen. Moreover, the secondary antibodies Alexa Fluor 594 (ab150080) and Alexa Fluor 488 (ab150113) were purchased from Abcam.

### Co-culture with spheroids to evaluate MXene@RGD’s ability to target tumors

To evaluate the tumor-targeting ability of MXene modified with RGD peptides, MXene@RGD was co-cultured with the spheroid. First, HeLa, CT-26, and MSCs were cultured for 2 d in LabSphero to form spheroids. Then, MXene solution and MXene@RGD solution were diluted to concentrations of 100 and 200 μg/ml with a growth medium, respectively. The spheroid obtained on the 2 d of culture was brought down by centrifugation at 500 rpm for 2 min, and the supernatant was removed and then replaced with MXene and MXene@RGD solutions prepared in advance. The spheroid was then suspended in an orbital shaker (RK-1D, ALLforLAB, Republic of Korea) at 50 rpm for 30 min while shaking. The evenly suspended spheroid was incubated for 6 h at 37 °C. After that, centrifugation at 500 rpm for 2 min, followed by washing with growth medium, was repeated 3 times.

### SEM imaging and EDS analysis of spheroids containing MXene@RGD

Spheroids were analyzed through scanning electron microscopy (SEM; SU3800, Hitachi) and energy-dispersive x-ray spectroscopy (EDS) to confirm MXene@RGD attachment to the cell surface. Prior to analysis, spheroids incubated with MXene and MXene@RGD solutions were attached to gelatin-coated coverslips. They were fixed with a 2.5% glutaraldehyde solution (G6257, Sigma-Aldrich, Switzerland), incubated overnight at 4 °C. Then, they were washed with PBS. Then, for SEM, spheroids were dehydrated using 50%, 75%, 95%, 95%, and 95% EtOH. Each concentration of EtOH was incubated for 10 min at room temperature. It was dried overnight in a hood at room temperature. The dried spheroid sample was coated with platinum sputtering for SEM imaging. Elemental analysis and mapping of spheroid surfaces using EDS and SEM imaging showed the distribution of MXene@RGD attached to spheroids.

### In vitro hemolysis analysis

Sheep blood (898890, Republic of Korea) was purchased from Biozoa Biological Supply for hemolysis analysis. To separate red blood cells (RBCs) in the blood, the blood was centrifuged at 10,000 rpm for 15 min. After that, the supernatant was removed and dispersed again in PBS. The separated RBCs were dispersed in MXene solution and MXene@RGD solution, respectively. RBCs were dispersed in PBS as a negative control group and in DW as a positive control group. The group containing RBCs was cultured at 450 rpm and 37 °C for 1 h. Moreover, after centrifugation at 10,000 rpm for 15 min, the supernatant was collected. The supernatant was measured for absorbance at 577 nm using a microplate reader. The following equation calculated the hemolysis rate according to the measured absorbance value:

$$\text{Hemolysis}\;\left(\%\right)=\frac{D_{t}-D_{\mathrm{nc}}}{D_{\mathrm{pc}}-D_{\mathrm{nc}}}\times 100.$$ (2)

*D_t_* is the sample tested, *D_nc_* is the negative control sample, and *D_pc_* is the positive control sample.

### Cytotoxicity test of MXene@RGD

To test the cytotoxicity of MXene@RGD, the (3-(4,5-dimethylthiazol-2-yl)-2,5-diphenyltetrazolium bromide (MTT) assay was used. MTT Assay Kit (Cell Proliferation) (ab211091, USA) was purchased from Abcam. First, HeLa, CT-26, and MSCs were seeded at 5,000 cells/well in 96-well plates. MXene and MXene@RGD solution were diluted with growth medium to 100 and 200 μg/ml, respectively. Each of the 3 types of cells was cultured in an unsampled growth medium and a growth medium diluted with MXene and MXene@RGD for 12, 24, and 36 h, respectively. After that, the medium was replaced with a solution mixed with 50 μl of serum-free media and 50 μl of MTT agent and incubated at 37 °C for 3 h. After replacement with 50 μl of MTT solvent, the plates were incubated at 50 rpm for 15 min using an orbital shaker in the dark. The absorbance was measured at 590 nm through a microplate reader.

### In vivo tumor reduction study

The animal experiments were approved by the Chonnam National University Medical School Institutional Animal Care and Use Committee (IACUC; Project CNU IACUC-H-2024-23), Republic of Korea. All of the animal experiments were conducted with relevant guidelines and regulations as per the university rules. Five-week-old BALB/c white mice were purchased from Orient Bio Inc., Republic of Korea. Subcutaneous colorectal tumors were induced on the right flanks of the mice. Briefly, 1 × 10^6^ cells were injected into the right flanks of the mice. As the tumor size reached 100 mm^3^, all of the animals were randomly divided into 5 different groups. The 5 different groups are PBS, MXene, MXene@RGD, MXene + laser, and MXene@RGD + laser. To each group except the PBS group, a MXene concentration of about 12 mg/kg (about 300 μg/mice) was injected constantly intravenously. After 24 h of injection, the animals were exposed to an irradiation of 1.5 W/cm^2^ of 808-nm NIR laser for 5 min. The temperature on the surface of the tumor was recorded using an infrared Avio IR camera (Yokohama, Japan), and the results were analyzed using the Thermomovie software. The tumor volume was measured using a vernier caliper on every alternate day. The tumor volume was measured as (length × width^2^/2) along with the body weight for 21 d. On the final day, the tumors along with the spleen were isolated and analyzed for tumor size and splenomegaly. Both tumor and spleen weights were recorded and analyzed.

### Immunohistochemistry study

The tested animals were euthanized, and the tumor tissues were collected. For immunohistochemistry, 10-μm sections were cut using a paraffin embedding system. Ki-67 and terminal deoxynucleotidyl transferase dUTP nick end labeling (TUNEL) assay were performed using these same sectioned slides. For the analysis of Ki-67, Ki-67 (D3B5) Rabbit mAb (Alexa Fluor 488 Conjugate) was used. The tissue sections were counterstained using DAPI to visualize the nucleus. Similarly, the TUNEL assay was performed as per the user guidelines mentioned in the DeadEnd Fluorometric TUNEL system (Promega Korea). The slides were analyzed using a confocal microscope to obtain 488-nm signals that correspond to Ki-67 Alexa Fluor 488 and TUNE-positive signals.

### Lung metastasis analysis

On the 21st day after the treatment, the lungs were injected with India ink intratracheally to monitor the metastatic lesions. Briefly, 1 to 2 ml of India ink was injected, and the tracheal puncture was blocked to ensure the complete reaction of the ink inside the lungs. Then, the lungs were pulled out together with the heart intact and dipped into Fekete’s solution. The stained tissues were fixed using Fekete’s solution for the analysis of the moderate white lesions on the lungs. Each nodule was counted both on the anterior and posterior parts of the lungs to calculate the metastatic nodules.

### Statistical analysis

Quantitative results were displayed in the form of mean ± standard deviation. One-way analysis of variance (ANOVA) and 2-way ANOVA of Šídák’s multiple-comparisons test were used to identify significant differences between experimental groups. *P < 0.05, **P < 0.01, ***P < 0.001, and ****P < 0.0001.

## Results and Discussion

### Characterization of MXene according to particle size

With the great development of nanotechnology in recent years, nanomaterials are also being used in research related to cancer treatment [9]. At the nanoscale, materials derived from bulk often exhibit altered composition and shape, and their physical and chemical properties are significantly changed compared to those in the bulk state [10]; their characteristics include the improvement of drug loading capacity due to large surface area [30], cell barrier passage, and enhanced permeability maintenance effect. We conducted experiments in comparison with microparticles to show the therapeutic efficacy of MXene nanoparticles at the beginning. In this study, surface-modified MXene nanoparticles conjugated with RGD peptides were synthesized for targeted delivery to tumor cells. Among the various 2D nanomaterials used in biomedical applications, MXene has garnered attention for its potential in tumor therapeutic interventions [31]. Firstly, MXene exhibits superior biodegradability and biocompatibility compared to graphene [32]. Secondly, the hydrophilic nature of the distal groups of MXene facilitates its stable dispersion under physiological conditions, distinguishing it from other 2D materials [33]. Moreover, the abundant surface functional groups on MXene offer ample opportunities for functionalization, including the loading of therapeutic agents [34]. Furthermore, MXene demonstrates promise in tumor treatment due to its high photothermal conversion efficiency. This is attributed to its potential as an effective therapeutic agent, leveraging its outstanding optical and thermal characteristics for tumor treatment modalities such as PTT, sonodynamic therapy, and chemodynamic therapy. Nonetheless, challenges persist in accurately targeting tumors. Previous studies on MXene nanoparticles for tumor treatment have predominantly focused on tumor accumulation and the passive EPR effect. The nonspecific distribution of MXene nanoparticles in vivo presents challenges, as it may inadvertently damage healthy tissue. Thus, addressing these limitations through the development of modified MXene is imperative.

**Fig. 2.**
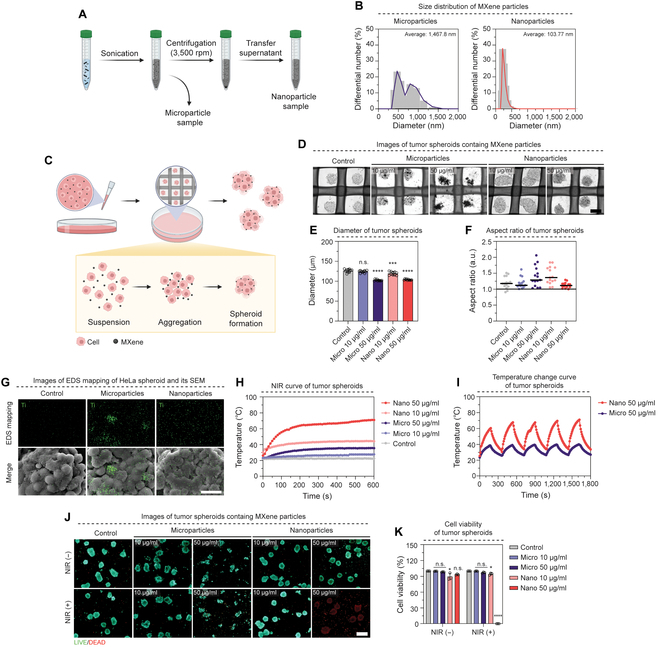
Characterization of MXene according to particle size. (A) A schematic diagram of MXene particle size control. (B) Dynamic light scattering (DLS) graph of MXene particle size distribution. (C) A schematic diagram of fabrication of tumor spheroids containing MXene particles. (D) Optical imaging of MXene spheroids for 2 d with different MXene concentrations (×200). (E) Diameter graph of tumor spheroids containing MXene. (F) Aspect ratio graph of tumor spheroids containing MXene. (G) Energy-dispersive x-ray spectroscopy (EDS) mapping and scanning electron microscopy (SEM) images of tumor spheroids with MXene (×2,000). (H) The temperature change curve of tumor spheroids with different MXene concentrations under irradiation with a laser power density (1.50 W/cm^2^) for 10 min. (I) Temperature change curve for the laser on–off cycle of tumor spheroids containing MXene. Heating and cooling for 3 min, a total of 5 cycles (808 nm, 1.50 W/cm^2^). (J) LIVE/DEAD staining images before and after the NIR laser irradiation of tumor spheroids for 10 min (green: live cells; red: dead cells) and (K) quantification of cell viability in each group. Scale bars: (D) 100, (G) 20, and (J) 200 μm. All data represent mean ± SD (*n* = 3). *P < 0.05, ***P < 0.001, and ****P < 0.0001. The symbol * indicates comparisons with a control group.

To investigate the potential impact of MXene particles on tumor cell death, we first characterized the distinct properties of MXene through EDS analysis and ultraviolet–visible spectroscopy (Fig. S1). MXene flakes were manipulated to achieve micro-sized and nano-sized particles through 1-h sonication and 1-h centrifugation at 3,500 rpm (Fig. 2A). DLS measurements indicated average particle sizes of 1,467 and 103.7 nm for the microparticle and nanoparticle groups, respectively (Fig. 2B). These findings set the stage for our investigation into the integration of MXene into tumor models. Tumor spheroids, chosen for their ability to replicate the intrinsic properties of cells and the characteristics of tissue units, were used. HeLa cells, commonly used in tumor research, were suspended in a cell culture medium containing MXene particles. During the 2-d culture period, the cells aggregated to form tumor spheroids containing MXene particles (Fig. 2C). Optical imaging revealed the presence of MXene particles in the microparticle group, displaying an uneven distribution, while a uniform distribution of MXene was observed throughout the spheroids in the nanoparticle group (Fig. 2D). The diameter of the tumor spheroids, including MXene particles, was measured, showing values of 124.17 and 102.45 μm at concentrations of 10 and 50 μg/ml for microparticles and 119.60 and 103.84 μm at concentrations of 10 and 50 μg/ml for nanoparticles, respectively (Fig. 2E). Notably, the analysis indicated a 1.05-fold decrease in diameter for the 10 μg/ml nanoparticle group and a 1.2-fold decrease for both the 10 and 50 μg/ml microparticle and nanoparticle groups compared to that of the control group. The aspect ratio was assessed to confirm the uniformity of tumor spheroid generation (Fig. 2F), demonstrating that the aspect ratio of the 10 μg/ml microparticle group and the 50 μg/ml nanoparticle group was similar to that of the control group approaching a value of 1.0. These findings suggest the successful integration of MXene particles into the tumor spheroid, acting as focal points for aggregation. Furthermore, analysis using EDS and mapping confirmed the distribution of MXene in cells (Fig. 2G and Fig. S2), revealing clumped titanium (Ti) element distribution in tumor spheroids for the microparticle group, whereas an even distribution of Ti elements was observed in the nanoparticle group. These results demonstrate the successful integration of MXene into tumor spheroids.

The photothermal effect was assessed by irradiating tumor spheroids containing MXene particles with NIR lasers. Before the experiment, MXene particles were introduced into tumor cells cultured in a 2D environment, and their photothermal properties were measured (Fig. S3). Tumor spheroids containing MXene were subjected to irradiation with an 808-nm NIR laser at an intensity of 1.5 W/cm^2^ for 10 min at different concentrations for microparticle and nanoparticle groups (Fig. 2H and Fig. S4A). Results indicated that the microparticle group reached a temperature of 27.7 °C at a concentration of 10 μg/ml and 35.5 °C at a concentration of 50 μg/ml, failing to exceed 40 °C. Conversely, the nanoparticle group reached temperatures exceeding 40 °C, recording a temperature of 44.2 °C at a concentration of 10 μg/ml and 71.0 °C at a concentration of 50 μg/ml. Notably, the 50 μg/ml nanoparticle group achieved a temperature approximately 3 times higher than that of the control group, demonstrating a superior photothermal conversion effect.

Furthermore, temperature change curves during the laser on–off cycles showed a similar increasing trend for both groups in 5 repetitions (Fig. 2I). However, the maximum temperature difference between the nanoparticle and microparticle groups was approximately 1.7-fold, indicating enhanced photothermal properties in the nanoparticle group. As a result of calculating the photothermal conversion efficiency, it was 2.83 times higher in the nanoparticle group at 40.21% compared to 14.20% in the microparticle group (Fig. S4B). Subsequent assessment of apoptosis via LIVE/DEAD staining of irradiated tumor spheroids using an NIR laser validated the presence of dead cells in the nanoparticle group at a concentration of 50 μg/ml (Fig. 2J).

Additionally, the cell viability of tumor spheroids pre- and post-NIR irradiation was quantified (Fig. 2K). Prior to NIR laser irradiation, cell viability was measured at 99.68% and 98.12% for microparticle concentrations of 10 and 50 μg/ml, respectively, and 89.44% and 93.32% for nanoparticle concentrations of 10 and 50 μg/ml, respectively. Viability exceeded 80% in all groups, indicating no cytotoxicity. However, after NIR laser irradiation, the microparticle group exhibited cell viabilities of 99.80% and 96.95% at concentrations of 10 and 50 μg/ml, respectively, while the nanoparticle group showed no viable cells. These findings suggest the superior photothermal effect and tumor-killing ability of MXene nanoparticles.

### Synthesis and characterization of MXene@RGD

MXene can act as a better photothermal agent than other materials, such as gold, silver, and graphene. First, MXene, as a photothermal agent, has a wider light absorption range than other materials. The light absorption spectra of gold and silver span visible and some NIR regions, with graphene in the NIR-I band in visible light, with reduced efficiency in NIR-II [35,36]. However, MXene has a light absorption range from visible light to NIR-II and has a wide and continuous wavelength absorption [37]. Second, MXene has excellent thermal conductivity. Gold and silver have a photothermal conversion temperature of about 40 to 55 °C, and graphene has a photothermal conversion temperature of about 40 to 60 °C. MXene appears in a wider temperature range of about 45 to 70 °C, which can contribute to efficiently optimizing the concentration of the MXene solution [38–40]. As the photothermal conversion temperature is high, effective photothermal treatment can be performed at a low concentration. Third, modification is easy through multifunctionality due to the surface functional groups of MXene [41]. Because surface chemical transformation is easy, functionality can be controlled, and various biomedical applications such as drug delivery and diagnostic imaging are possible in addition to photothermal treatment [42].

**Fig. 3.**
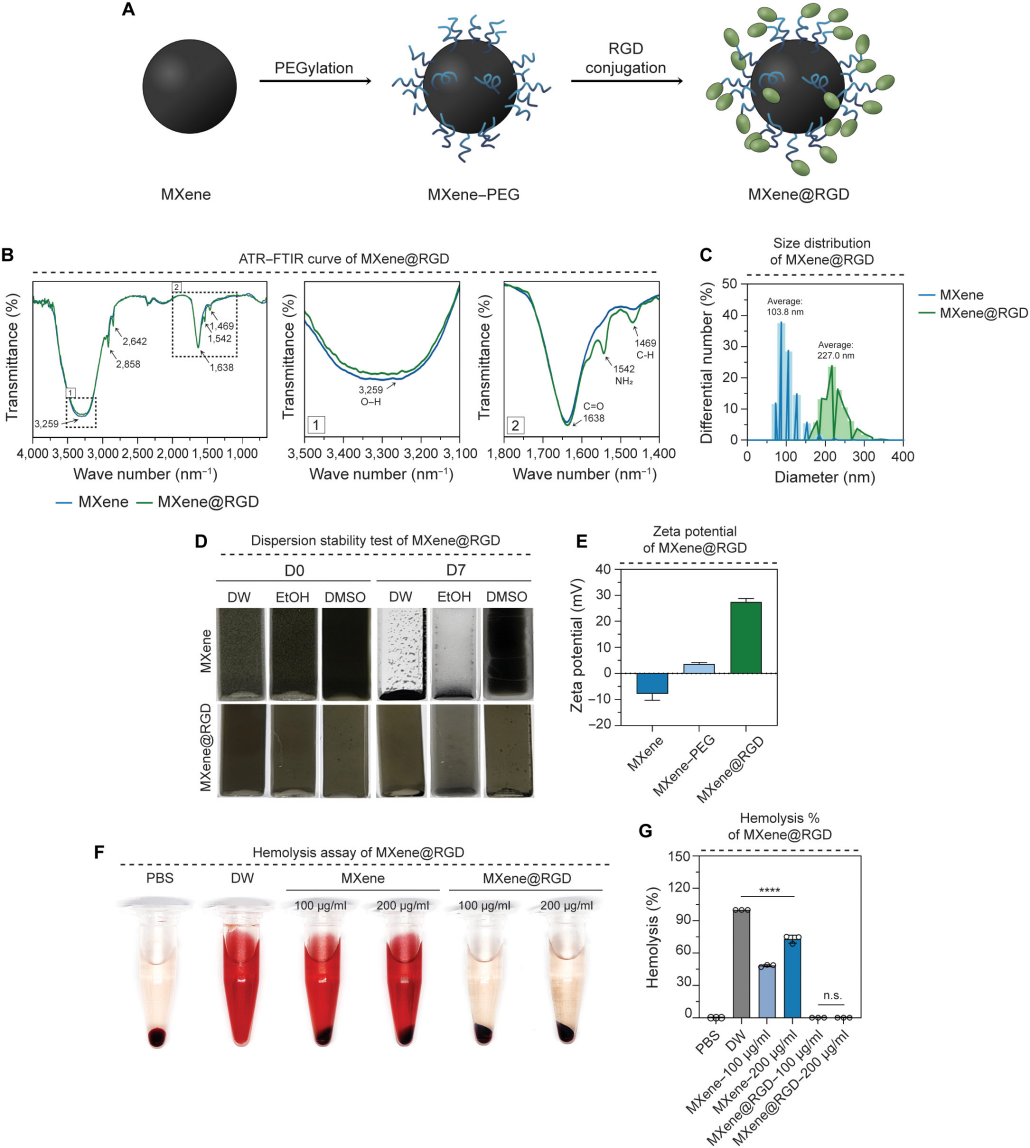
Synthesis of MXene modification and characterization. (A) Schematic diagram of the conjugation of poly(ethylene glycol) (PEG) and RGD peptide on the surface of MXene nanoparticles. (B) Attenuated total reflectance–Fourier transform infrared (ATR–FTIR) spectra of MXene and MXene@RGD. (C) Dynamic light scattering (DLS) graphs according to particle distribution. (D) Distributed stability image after day 7. (E) Zeta potential graph. (F) Hemolysis analysis image according to concentration and (G) quantification of hemolysis% in each group. All data represent mean ± SD (*n* = 3). ****P < 0.0001. The symbol * indicates comparisons with the phosphate-buffered saline (PBS) group. DW, deionized water; EtOH, ethanol; DMSO, dimethyl sulfoxide.

Surface modification of MXene plays a crucial role in addressing the challenges related to the high temperature of MXene and its associated side effects during the treatment process. Our approach involved the sequential synthesis of PEG and RGD peptides using functional groups on the MXene surface to prepare MXene@RGD (Fig. 3A and Fig. S5). The surface modification of MXene was confirmed through analysis using ATR–FTIR, which indicated the peaks at 1,542 cm^−1^ (–NH_2_) and 1,469 cm^−1^ (C–H), demonstrating the successful synthesis of PEG and RGD peptides along with the migration of 3,259 cm^−1^ (–OH) and 1,638 cm^−1^ (C=O) (Fig. 3B). Furthermore, the particle size distribution analysis via DLS revealed that the particle size of MXene@RGD increased from 103.8 to 227.0 nm (Fig. 3C), indicating the successful synthesis of MXene@RGD. The dispersion stability of the synthesized material is crucial for potential clinical trials or medical applications. Dispersion stability in DW, EtOH, and dimethyl sulfoxide was evaluated over 7 d (Fig. 3D). Zeta potential is one of the factors that shows an important influence on cell absorption or cell adhesion. Since the cell membrane is negatively charged, the more positively charged it is, the more it can interact with the cell membrane by electrostatic attraction. We measured the zeta potential to show the surface charge of unmodified MXene, MXene–PEG, and MXene@RGD. Zeta potential values showed −7.87 mV for unmodified MXene, 3.65 mV for MXene–PEG, and 27.475 mV for MXene@RGD (Fig. 3E). Therefore, MXene@RGD may act favorably on cell adhesion and cell absorption. It was observed that unmodified MXene was noticeably precipitated, while MXene@RGD exhibited higher dispersion stability, remaining evenly dispersed in the solvents. Moreover, hemolysis rates were assessed using RBCs in co-culture. Visual observation and quantification of the hemolysis rate through absorbance indicated that unmodified MXene exhibited hemolysis at 100 and 200 μg/ml concentrations, whereas the MXene@RGD group showed no significant hemolysis, similar to the negative control group (PBS) (Fig. 3F and G). Based on these results, we propose that the surface modification of MXene with RGD peptides enhances its stability in an in vivo environment.

### Targeting ability of MXene@RGD in tumor spheroids

**Fig. 4.**
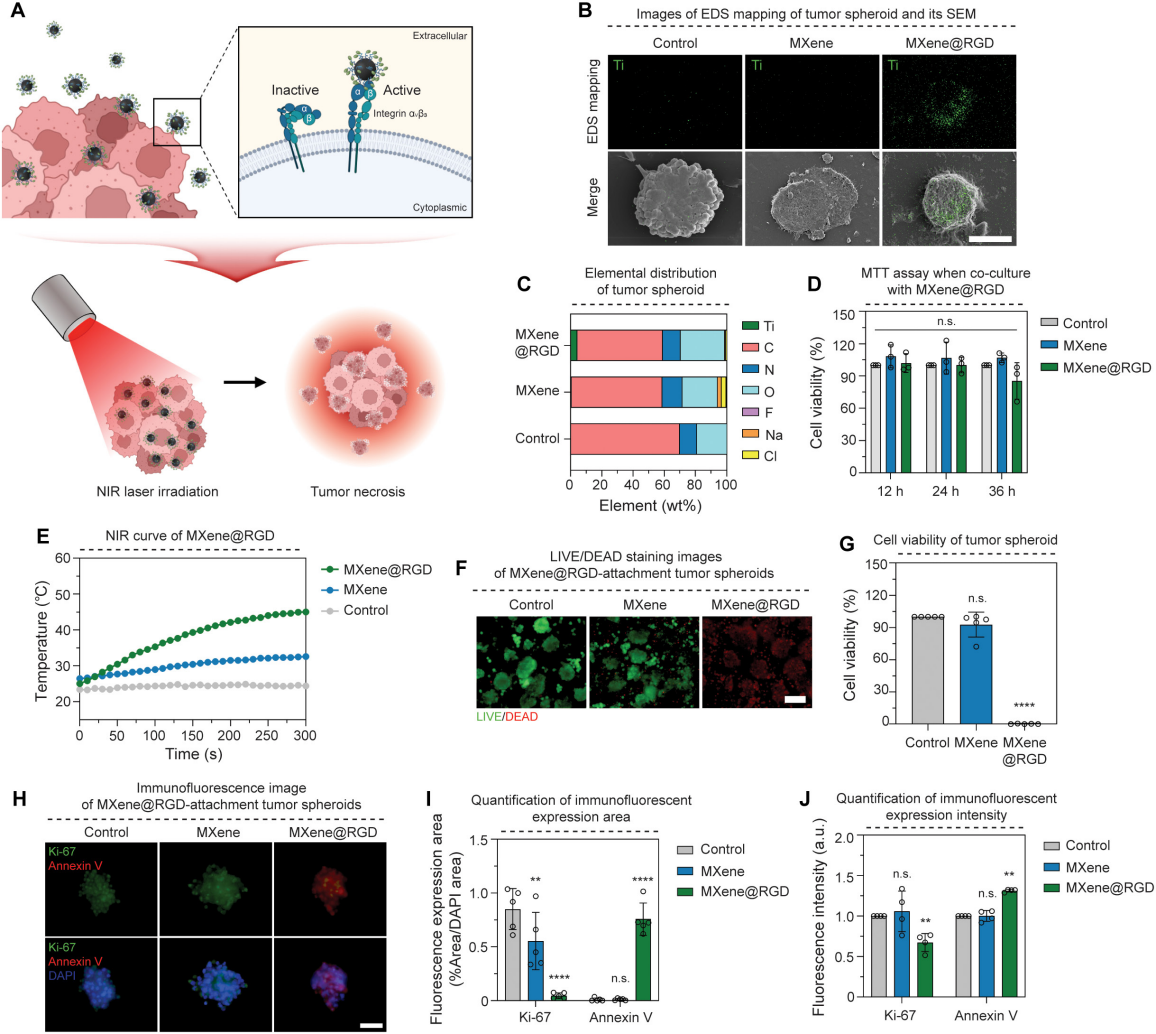
Targeting ability of MXene@RGD in tumor spheroids. (A) Schematic diagram of tumor spheroid attachment and apoptosis of MXene@RGD. (B) Ti element mapping and SEM image of tumor spheroids (×800). (C) EDS elemental analysis graph of tumor spheroids. (D) Cell viability graph by (3-(4,5-dimethylthiazol-2-yl)-2,5-diphenyltetrazolium bromide (MTT) analysis. (E) The temperature change curve of tumor spheroids with a laser power density (1.50 W/cm^2^) for 5 min. (F) LIVE/DEAD staining images after the NIR laser irradiation of tumor spheroids for 5 min (×200) (green: live cells; red: dead cells) and (G) quantification of cell viability in each group. (H) Immunofluorescent staining image of tumor spheroids (×630); green: Ki-67; red: annexin V; blue: 4′,6-diamidino-2-phenylindole (DAPI). (I) Quantification of immunofluorescent expression area and (J) expression intensity. Scale bars: (B) 50, (F) 100, and (H) 40 μm. All data represent mean ± SD (*n* = 3 to 5). **P < 0.01 and ****P < 0.0001. The symbol * indicates comparisons with a control group.

An investigation into the tumor-targeting capability of the synthesized MXene@RGD was conducted. Immunofluorescence staining was employed to confirm the expression of integrin α_v_β_3_, a tumor-specific target in this study (Fig. S6). Tumor spheroids were prepared in advance, and a diluted form of MXene@RGD was introduced into the culture medium (Fig. 4A). Following co-cultivation and subsequent washing, the distribution of Ti elements was confirmed through SEM photographs and EDS mapping images (Fig. 4B and Table). The EDS mapping revealed that while the unmodified MXene group did not exhibit Ti element distribution due to washing, the MXene@RGD group displayed Ti element distribution on the surface of the tumor spheroid. Additionally, the percentages of Ti elements in the unmodified MXene group and MXene@RGD group were found to be 0.69 and 4.44 wt%, respectively, as determined by EDS elemental analysis (Fig. 4C), indicating a substantial difference of approximately 6.44 times. This finding suggests the attachment of MXene@RGD to the tumor spheroid. MTT analysis indicated that when co-cultured with tumor cells for 12, 24, and 36 h, cell viability exceeded 80% in all instances, demonstrating biocompatibility (Fig. 4D). Furthermore, compared to unmodified MXene, MXene@RGD exhibited the ability to adhere to tumor cells without displaying cytotoxic effects. The tumor spheroids from each group were subsequently irradiated with NIR laser at a power density of 1.5 W/cm^2^ for 5 min (Fig. 4E). The unmodified MXene recorded a temperature of 32 °C, lower than the body temperature, whereas MXene@RGD reached 45 °C, representing a temperature rise approximately 1.3 times higher. LIVE/DEAD staining revealed a significant number of dead cells in the MXene@RGD group (Fig. 4F). Additionally, cell viability was remarkably low, indicating targeted tumor cell death (Fig. 4H). Immunofluorescence staining of the NIR-laser-irradiated tumor spheroid demonstrated a predominance of the tumor cell proliferation factor Ki-67 in the control and unmodified MXene groups, whereas annexin V, a cell necrosis factor, was more prevalent in the MXene@RGD group (Fig. 4G). The analysis of the fluorescence expression area showed an 11-fold decrease in Ki-67 and a 70-fold increase in annexin V in comparison to unmodified MXene (Fig. 4I). Moreover, the fluorescence intensity measurements revealed a 1.31-fold increase in annexin V in comparison to unmodified MXene (Fig. 4J). Consequently, the results indicated that in the MXene@RGD group, annexin V, as a cell necrosis factor, was more prevalent than in the unmodified MXene group, indicating tumor death due to effective tumor targeting. Therefore, owing to its effective tumor cell adhesion, the surface-modified MXene@RGD exhibited enhanced photothermal conversion and induced greater tumor cell death compared to unmodified MXene.

**Table.** Summary of FTIR data reported as wave number (cm^−1^)

Functional groups	Compounds	MXene@RGD
O–H stretch	ND	3,259
C≡N	ND	2,858
C≡C	ND	2,642
C=O	Carboxylic acid	1,638
–NH_2_	Amino group	1,542
C–H	ND	1,469

ND, no data

### Selective targeting ability of other tumor spheroids and normal cell spheroids MXene@RGD

**Fig. 5.**
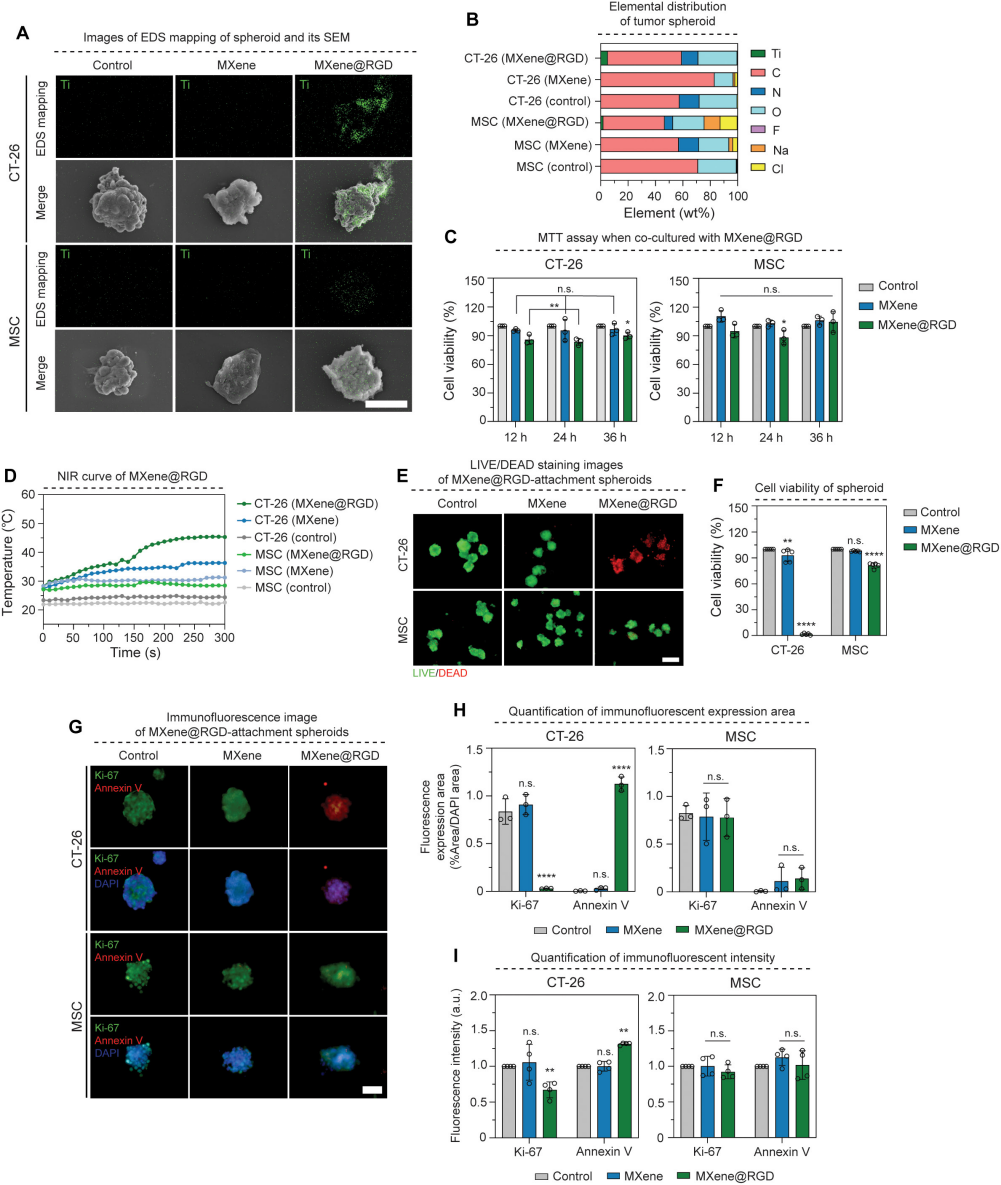
Selective targeting ability of other tumor spheroids and normal cell spheroids MXene@RGD. (A) Ti element mapping and SEM image of tumor spheroids (×800). (B) EDS elemental analysis graph of tumor spheroids. (C) Cell viability graph by MTT analysis. (D) The temperature change curve of tumor spheroids with a laser power density (1.50 W/cm^2^) for 5 min. (E) LIVE/DEAD staining images after the NIR laser irradiation of tumor spheroids for 5 min (×200) (green: live cells; red: dead cells) and (F) quantification of cell viability in each group. (G) Immunofluorescent staining image of tumor spheroids (×630); green: Ki-67; red: annexin V; blue: DAPI. (H) Quantification of immunofluorescent expression area and (I) expression intensity. Scale bars: (A) 50, (E) 100, and (G) 40 μm. All data represent mean ± SD (*n* = 3 to 5). *P < 0.05, **P < 0.01, and ****P < 0.0001. The symbol * indicates comparisons with a control group.

The overexpression of integrin α_v_β_3_ in tumor cells makes it a common marker. To investigate the potential attachment of MXene@RGD to tumor cells other than HeLa cells, CT-26 cells, which are colon tumor cells, were employed. Additionally, MSCs were used as normal cells to assess selective adhesion. Co-culturing of each cell type with MXene@RGD followed by EDS mapping revealed substantial attachment of MXene@RGD to CT-26 spheroids, similar to the results obtained with HeLa cells (Fig. 5A). Conversely, MSC spheroids exhibited lower Ti element expression from the attached MXene@RGD. Quantification via EDS elemental analysis demonstrated an approximately 2.7-fold greater adhesion of MXene@RGD to tumor spheroids, with Ti element distributions of 5.30 wt% in CT-26 cells and 1.99 wt% in MSCs (Fig. 5B). Biocompatibility testing using MTT analysis indicated a cell survival rate of 80% for both tumor and normal cells co-cultured with the samples for 12, 24, and 36 h (Fig. 5C). To assess selective tumor cell death, the treated tumor and normal spheroids were irradiated with a 1.5 W/cm^2^ NIR laser for 5 min (Fig. 5D). The final temperatures of the unmodified MXene group were similar in CT-26 and MSC spheroids (36.3 and 31.2 °C, respectively), whereas the MXene@RGD group exhibited a 1.5-fold difference in final temperatures (45.3 °C in CT-26 and 28.5 °C in MSC). LIVE/DEAD staining illustrated effective tumor spheroid destruction by MXene@RGD in CT-26 spheroids (Fig. 5E). This was further supported by cell viability quantification, with the MXene@RGD group showing 1.66% cell viability in CT-26 spheroids, while MSC spheroids exhibited 81.19% viability (Fig. 5F). Immunofluorescence staining showed high expression of annexin V, a cell necrosis factor, in the CT-26 spheroids of the MXene@RGD group (Fig. 5G). Fluorescence expression area measurements revealed significant changes in unmodified MXene and MXene@RGD groups in CT-26 spheroids, with a 30-fold decrease in Ki-67 and a 38-fold increase in annexin V in the MXene@RGD group.

In contrast, similar fluorescence expression areas were observed for both unmodified MXene and MXene@RGD groups in the MSC spheroids (Fig. 5H and I). The results suggest that MXene@RGD selectively attaches to tumor cells with overexpression of integrin α_v_β_3_, resulting in cell death. Moreover, the observed effects across different tumor cell types demonstrate the potential of MXene@RGD for broader applications in tumor treatments.

Numerous investigations are underway to modify the surface properties of MXene nanoparticles to confer tumor selectivity. Essential considerations in designing clinically applicable antitumor agents encompass factors such as particle size, shape, stability in co-culture with cells, and targeting capabilities [43]. First, by manufacturing MXene nanoparticles to favor the EPR effect and evaluating their photothermal properties, they not only predicted EPR effects due to nanoscale but also showed improved photothermal effect and tumor cell death capacity due to their large surface area. Experimental findings demonstrated enhanced photothermal effects and tumor cell cytotoxicity attributed to the nanoparticles’ size and large surface area. Second, in order to introduce a target-oriented ligand, the MXene nanoparticles were surface-modified using PEG, a biocompatible polymer. Subsequently, the surface of MXene nanoparticles was modified with PEG, a biocompatible polymer, to introduce a target-specific ligand. Effective dispersion stabilization is crucial for clinical experimentation and medical applications. PEG, recognized for its stabilizing properties, has been extensively studied for PEGylation as a method for dispersion stabilization. Notably, this study revealed improved dispersion stability following PEGylation.

Furthermore, PEG aids in enhancing cellular interaction by serving as a spacer, creating gaps between particles and cells. Third, surface modification of MXene nanoparticles, including RGD peptide conjugation, has shown promising results in selectively targeting tumor cells. The engineered MXene@RGD demonstrates selective tumor cell eradication by targeting integrin α_v_β_3_, which is overexpressed in tumor cells. The guanidine group of arginine of MXene@RGD binds side by side to Asp218 of α_v_β_3_’s α-subunit to form a double salt bridge [44]. This enables the active targeting of tumors. This converts MXene nanoparticles from passive target orientation due to EPR effects to active target orientation and can serve as more improved antitumor drugs. Moreover, integrin α_v_β_3_, the target of RGD peptides, is known to be overexpressed in a wide array of tumor cells. Experiments utilizing different tumor cell types, including HeLa cells and CT-26 cells, suggest potential applicability across various tumor types, transcending specific tumor classifications.

### Tumor targeting and tumor killing of MXene@RGD in vivo

**Fig. 6.**
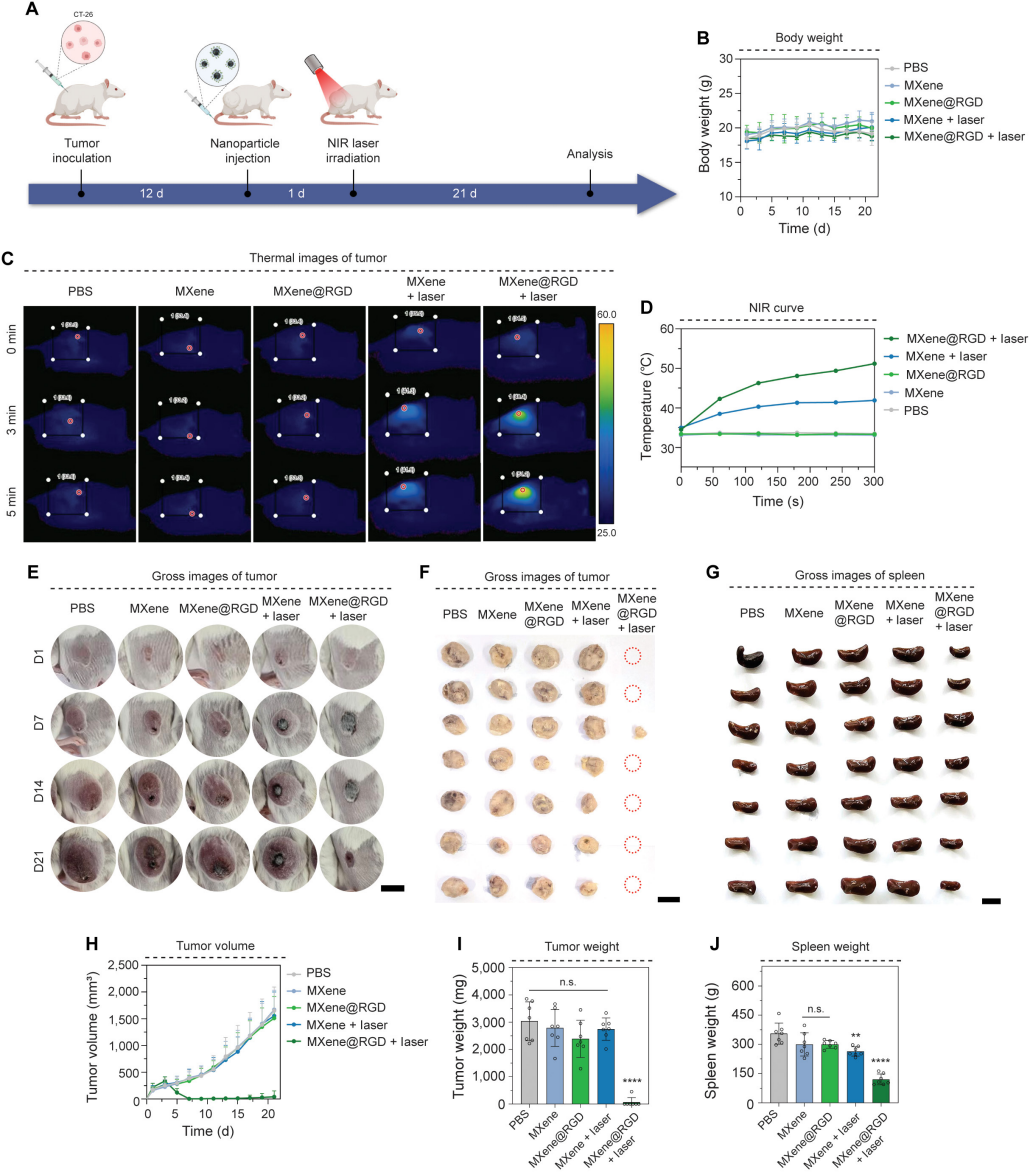
Tumor targeting and tumor killing of MXene@RGD in vivo. (A) Schematic diagram of in vivo injection experiments of MXene@RGD. (B) Weight change curve graph of mice. (C) NIR-irradiated thermal imaging images of mice. (D) The temperature change curve of tumor spheroids with a laser power density (1.50 W/cm^2^) for 5 min. (E) Gross images of mouse tumor for 21 d. (F) Tumor images of mice were taken 21 d later. (G) Gross images of the mice’s kidney. (H) Graph of the change in volume of tumor. (I) Graph of the change in weight of tumor. (J) Graph of the weight of the spleen. Scale bars: (E) 1, (F) 2, and (G) 1 cm. All data represent mean ± SD (*n* = 7). ****P < 0.0001. The symbol * indicates comparisons with a PBS group.

Previously, HeLa and CT-26 were used to show selective targeting and killing of various tumors. Among them, colorectal cancer is the third most frequently diagnosed disease in men and the second most frequently diagnosed disease in women, and the incidence rate is increasing in 2020, accounting for 10% of the global cancer incidence and 9.4% of cancer mortality [45,46]. In vivo experiments of MXene@RGD were conducted to treat these colorectal cancers. To elucidate the antitumor efficacy of MXene@RGD nanoparticles, the tumor volume was continuously monitored for 21 d (Fig. 6A). Weigh changes in mice over 21 d were measured and remained similar in all groups without significant reductions (Fig. 6B). After 5 min of NIR laser irradiation at the tumor site, the temperature of the other group without RGD was only 41.9 °C after 5-min laser irradiation, but the MXene@RGD + laser group achieved a photothermal effect with a maximum temperature rise to 51.2 °C, reaching a temperature 1.22 times higher than that of the MXene + laser group (Fig. 6C and D). This required the accumulation of MXene particles in the tumor region to increase heat when irradiated with the NIR laser, and it was clear that the accumulation of MXene particles was a major factor due to the targeting of the RGD peptide. In addition, visual images from mice revealed that the MXene@RGD + laser group exhibited a significant decrease in tumor volume compared to the other groups (Fig. 6E and F). This could be attributed very well to the targeting ability of RGD peptides to integrin, especially integrin α_v_β_3_, which is overexpressed on the CT-26 tumor cell surface. As a result of measuring tumor volume and weight, other groups showed no significant difference, while they definitively decreased in the MXene@RGD + laser group (Fig. 6H and I). It was clear that only the final group with laser had a significant effect on tumor reduction due to temperature rise.

Additionally, the spleen was isolated, and size changes were analyzed (Fig. 6G and J). There was a clear change in spleen size due to the inflammatory response that resulted in infiltration of immune cells and increased size [47]. However, the final group, the MXene@RGD + laser group, rapidly decreased in size and weight. This may be due to the reduced severity of other organs due to tumor reduction due to PTT. Finally, spleen weights were also measured, and the final group showed a significant reduction in spleen size and weight, indicating the efficacy of MXene@RGD + laser in reducing tumor severity.

### Tumor apoptosis and metastasis of MXene@RGD in vivo

**Fig. 7.**
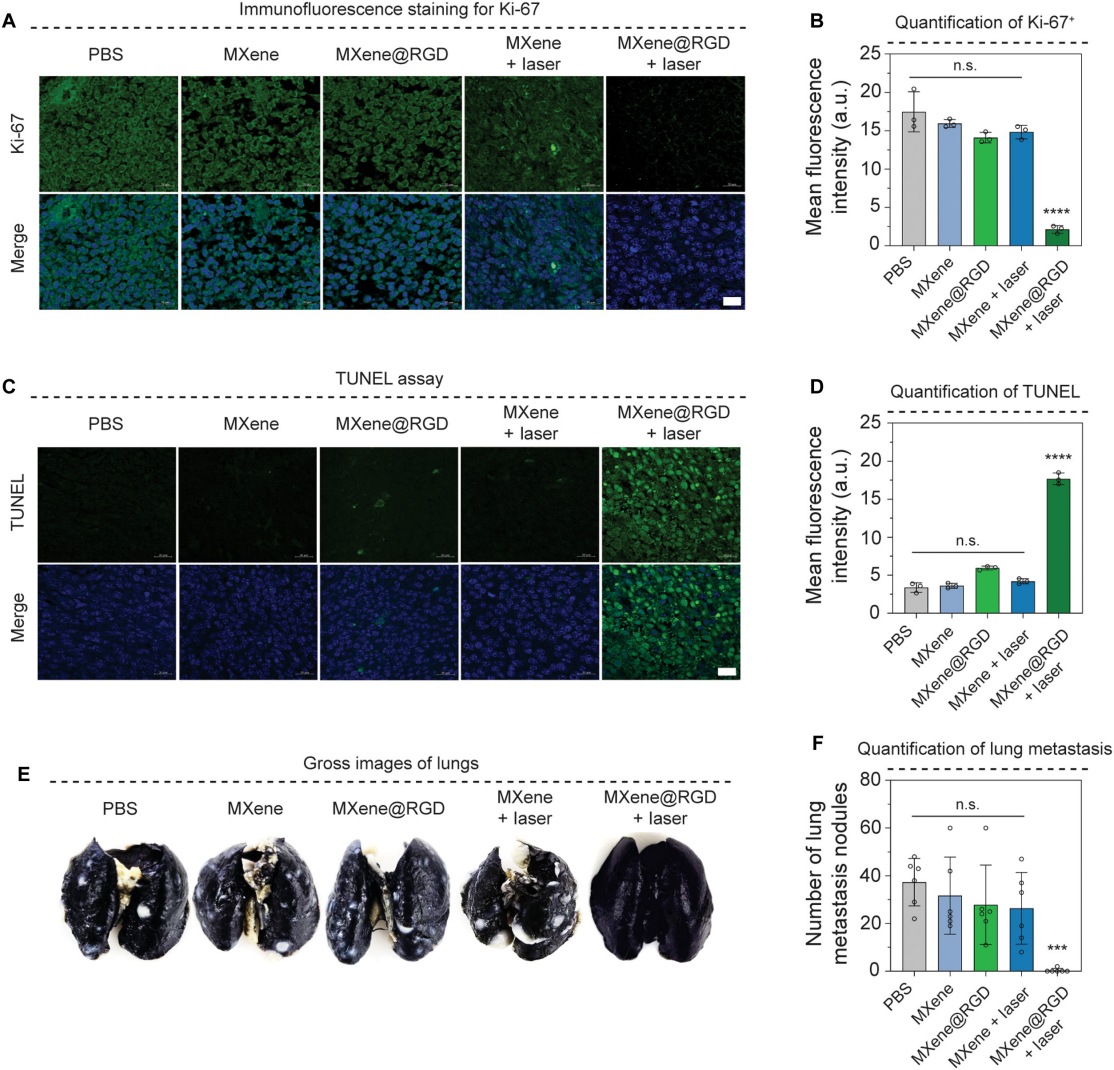
Tumor apoptosis and metastasis of MXene@RGD in vivo. (A) Immunofluorescence images of tumors for Ki-67. (B) Quantification graph of Ki-67 expression. (C) Images of the terminal deoxynucleotidyl transferase dUTP nick end labeling (TUNEL) analysis of the tumor. (D) Quantification graph for TUNEL assay. (E) Images of the lungs of mice. (F) Lung nodule graph for the lungs of mice. Scale bar: (A and C) 20 μm. All data represent mean ± SD (*n* = 3 to 6). ***P < 0.001 and ****P < 0.0001. The symbol * indicates comparisons with a PBS group.

Segmented tumor slides were analyzed for cell proliferation and cell death analysis. Staining of slides with Ki-67, a cell proliferation marker, revealed that the groups without laser irradiation showed green fluorescence of cell proliferation similar to that of the PBS group, but in the MXene@RGD + laser group, there was little green fluorescence due to decreased proliferation in tumor tissue (Fig. 7A). It showed signs contrary to those of the unmodified MXene + laser group. Quantification of green fluorescence revealed that the MXene@RGD + laser group exhibited 6.98-fold lower cell proliferation than the MXene + laser group (Fig. 7B). In addition, we performed TUNEL analysis to show cell death signals in tumor tissue (Fig. 7C). Here, we clearly showed the increase in green fluorescence, showing the presence of cell death signals in the tumor tissue of the MXene@RGD + laser treatment group, but in all other groups, no significant cell death occurred, demonstrating the tumor eradication efficacy of MXene@RGD + laser. The MXene@RGD + laser group exhibited 4.22 times higher apoptosis than MXene + laser (Fig. 7D). Then, 21 d later, we monitored the tumor’s metastasis to the distal organ. As tumor growth increased, the probability of the tumor reaching the distal organ, such as the lungs, was higher. While comparing the tumor volume graph (Fig. 7H), only the final group showed a rapid tumor reduction and almost complete tumor eradication. Identification the of Indian inked lung revealed distinct white nodules in all other groups except MXene@RGD + laser (Fig. 7E and F). This eventually occurs when the tumor migrates with the blood system and forms metastatic nodules in the lung, but cell migration decreased sharply as the MXene@RGD + laser group significantly eradicated the tumor on day 6, as shown in Fig. 6H. There were no significant differences in metastatic nodules between PBS, MXene, MXene@RGD, and MXene + laser. Finally, all separated organs, such as the liver, heart, kidney, lung, and spleen, were treated with nanoparticles and then tested by hematoxylin and eosin staining for damage to tissues (Fig. S7). There was no damage to tissues, and MXene and MXene@RGD particles were found to be highly biocompatible.

Our findings underscore the potential of MXene as an effective tumor therapeutic agent, attributing its prowess to its exceptional photothermal conversion properties and its successful targeting of tumors through surface modification utilizing RGD peptides. The multifunctional groups rendered by MXene present abundant possibilities for surface modifications, underscoring it as a promising platform for further advancements in targeted tumor treatment. This study highlights the importance of MXene in improving targeting ability and efficacy in tumor treatment, paving the way for further development in this field. Surface modification of MXene holds promise as a paramount tool for improved targeting and more effective tumor treatment.

## Conclusion

The recent investigation involved the development of RGD peptide-based surface-modified MXene nanoparticles for targeted tumor therapy. The nanoparticles, with their substantial surface area, exhibited heightened photothermal conversion capabilities and cytotoxicity compared to microparticles. Furthermore, the surface modification of MXene nanoparticles with RGD peptides enabled specific targeting of integrin α_v_β_3_, enhancing tumor selectivity. This modification resulted in significantly greater adhesion to tumor cells than to normal cells, leading to tumor cell death. The study highlights the potential for selective eradication of tumor cells while sparing healthy tissues through MXene surface modification, suggesting a promising approach for effective tumor-targeting treatment.

## Data Availability

The data are freely available upon request.
