# Supplementary material for: Optimizing the Surface Functionalization of Peptide–MXene Nanoplatforms to Amplify Tumor-Targeting Efficiency and Photothermal Therapy
Source: Biomater Res. 2025 May 26;29:0198. doi: 10.34133/bmr.0198 (PMC12104560; doi:10.34133/bmr.0198)
Supplement: Supplementary 1 — Figs. S1 to S7 [file bmr.0198.f1.docx]

**Supplementary Figures**

**
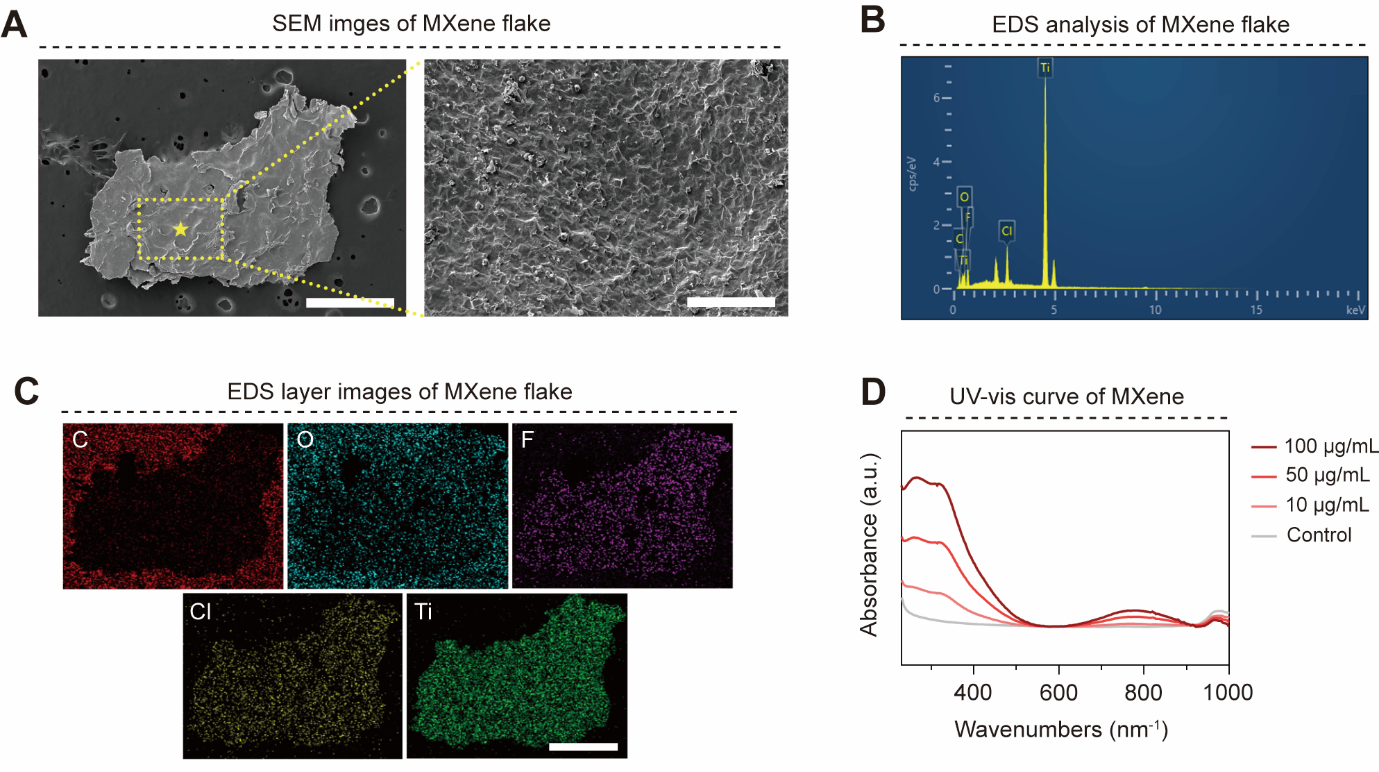
**

**Figure S1.** Characterization of MXene. (A) SEM image of MXene flake (X30) and zoom-in on a MXene flake surface (X1,00k). (B) EDS analysis of MXene flake. (C) Element mapping images of MXene flake (X30). (D) UV-vis absorbance spectra of the MXene solution according to particle size (Control: distilled water). Scale bar: (A) 1 mm (left), 25 μm (right), (C) 1 mm.


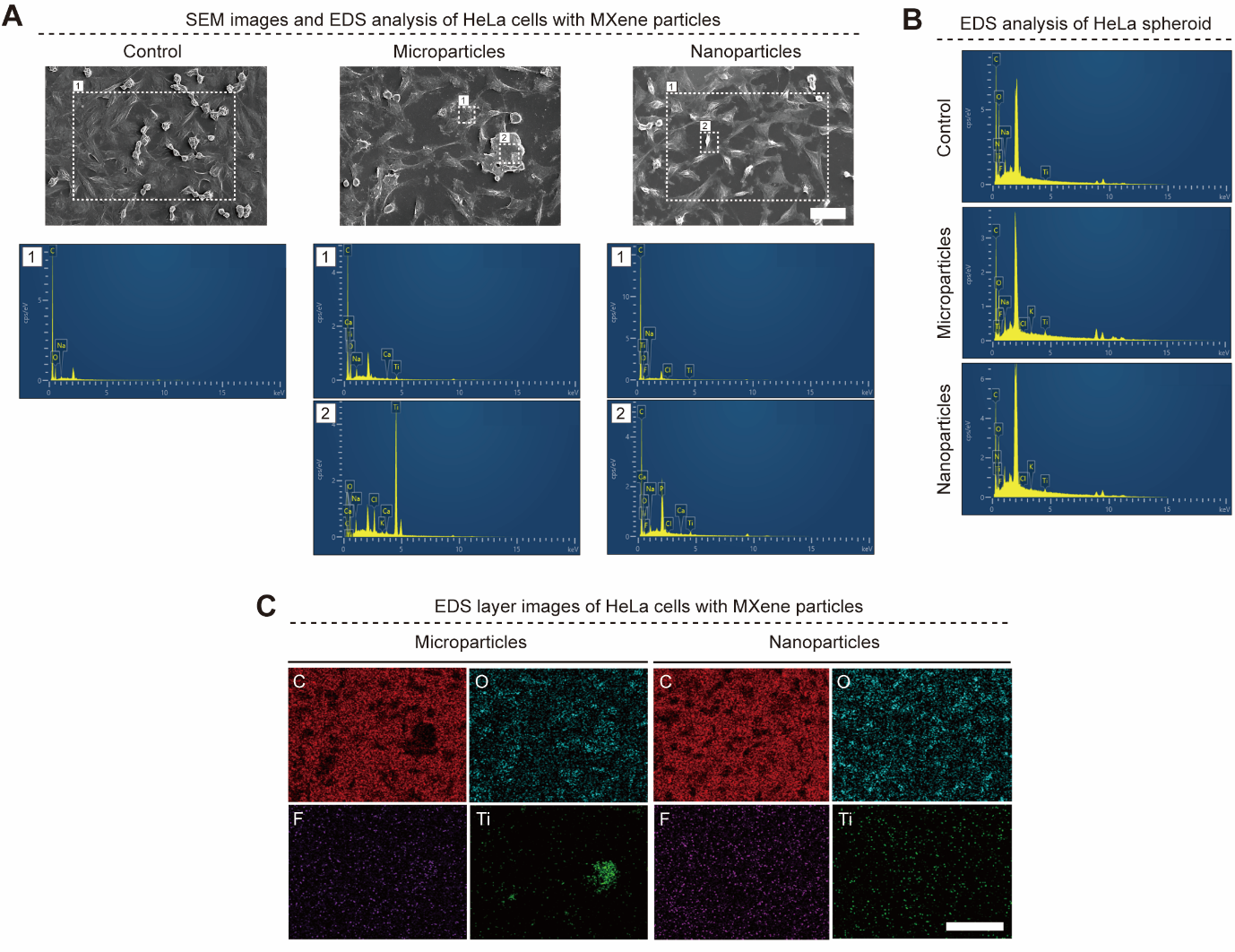


**Figure S2.** Element distribution of MXene according to particle size when tumor cells and spheroids. (A) SEM images and EDS analysis of tumor cells with MXene particles (X400). (B) EDS analysis of tumor spheroids with MXene particles. (C) Element mapping images of tumor cells with MXene particles (X400). Scale bar: (A) 50 μm, (C) 100 μm.


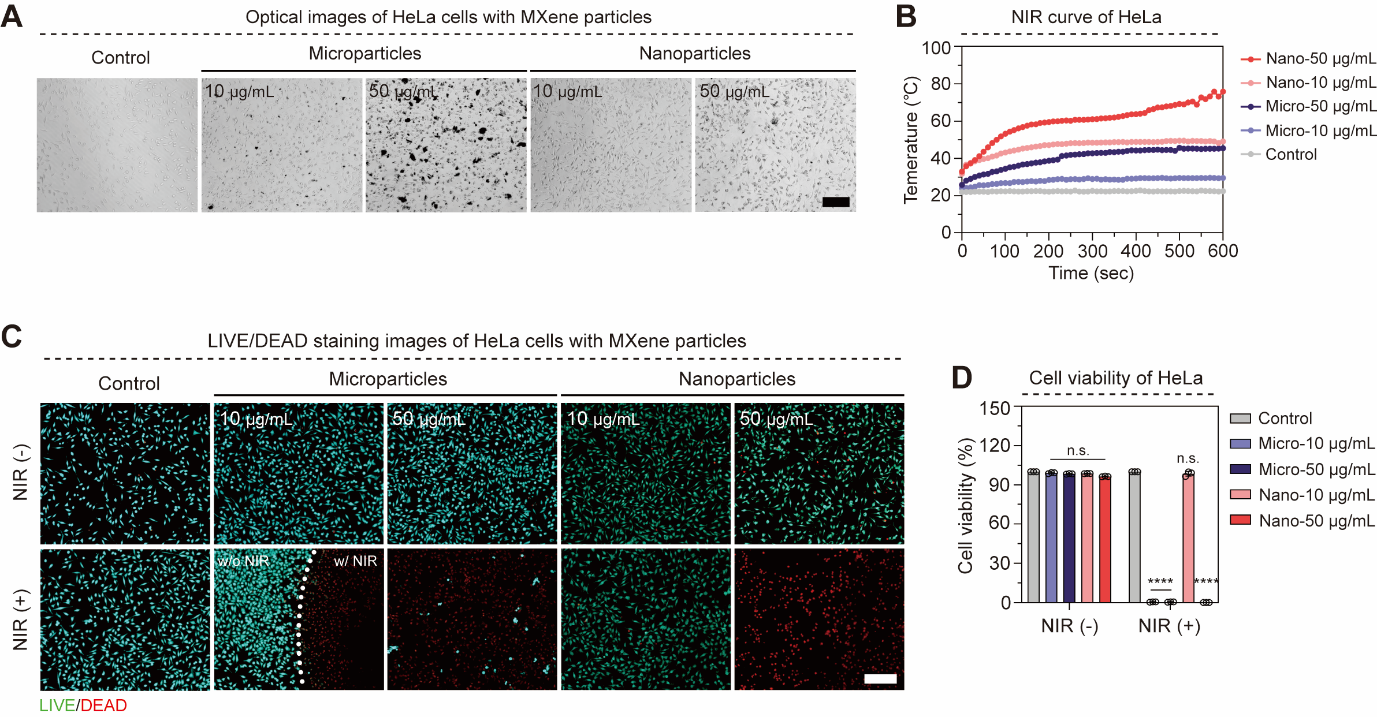


**Figure S3.** Photothermal properties of MXene according to particle size when tumor cells are cultured on TCP. (A) Optical images of tumor cells with MXene particles (X100). (B) Photothermal heating curve according to the concentration of MXene when irradiated with 808 nm laser power density (1.50 W/cm^2^) for 10 min. (C) LIVE/DEAD staining images before and after the NIR laser irradiation of tumor cells for 10 min (X100); green: live cells, red: dead cells. The white dashed line indicates laser irradiation boundaries. (D) Cell viability of tumor cells before and after NIR irradiation. Control: non-treat, tumor cell only. Scale bar: (A) 200 μm, (C) 200 μm. All data represent mean ± SD (n=3). ****p < 0.0001. The symbol * indicates comparisons with a control group.


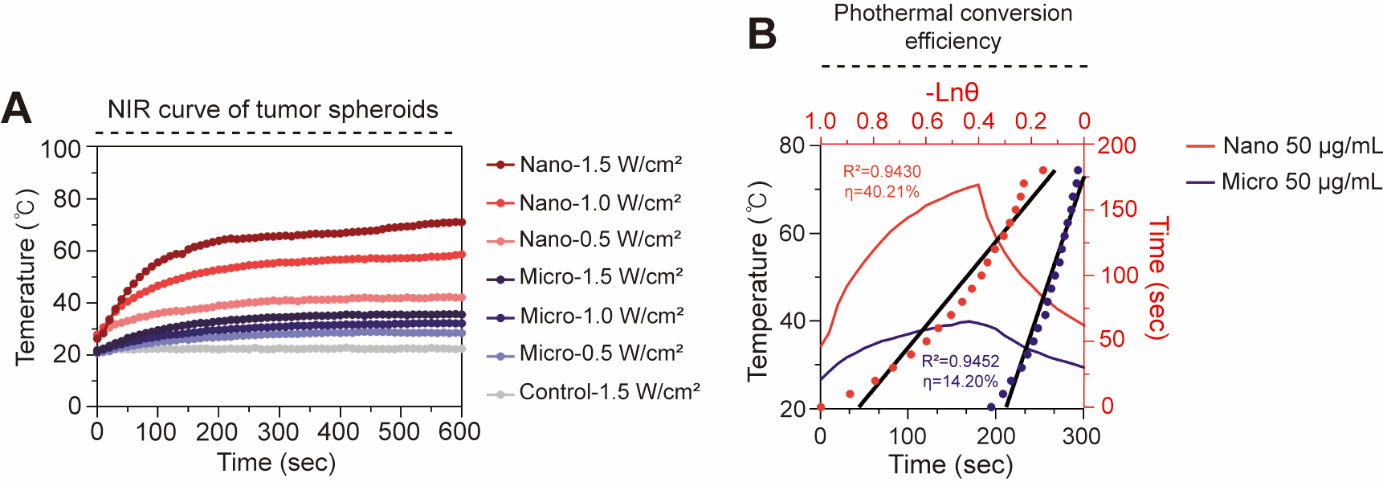


**Figure S4.** Photothermal heating curves and photothermal conversion efficiency for microparticles and nanoparticles. (A) The photothermal heating curve of tumor spheroids with MXene particles irradiated with different 808 nm laser power densities (0.50, 1.00, and 1.50 W/cm^2^) for 10 min (n=3). (B) Graph of photothermal conversion efficiency for microparticles and nanoparticles.


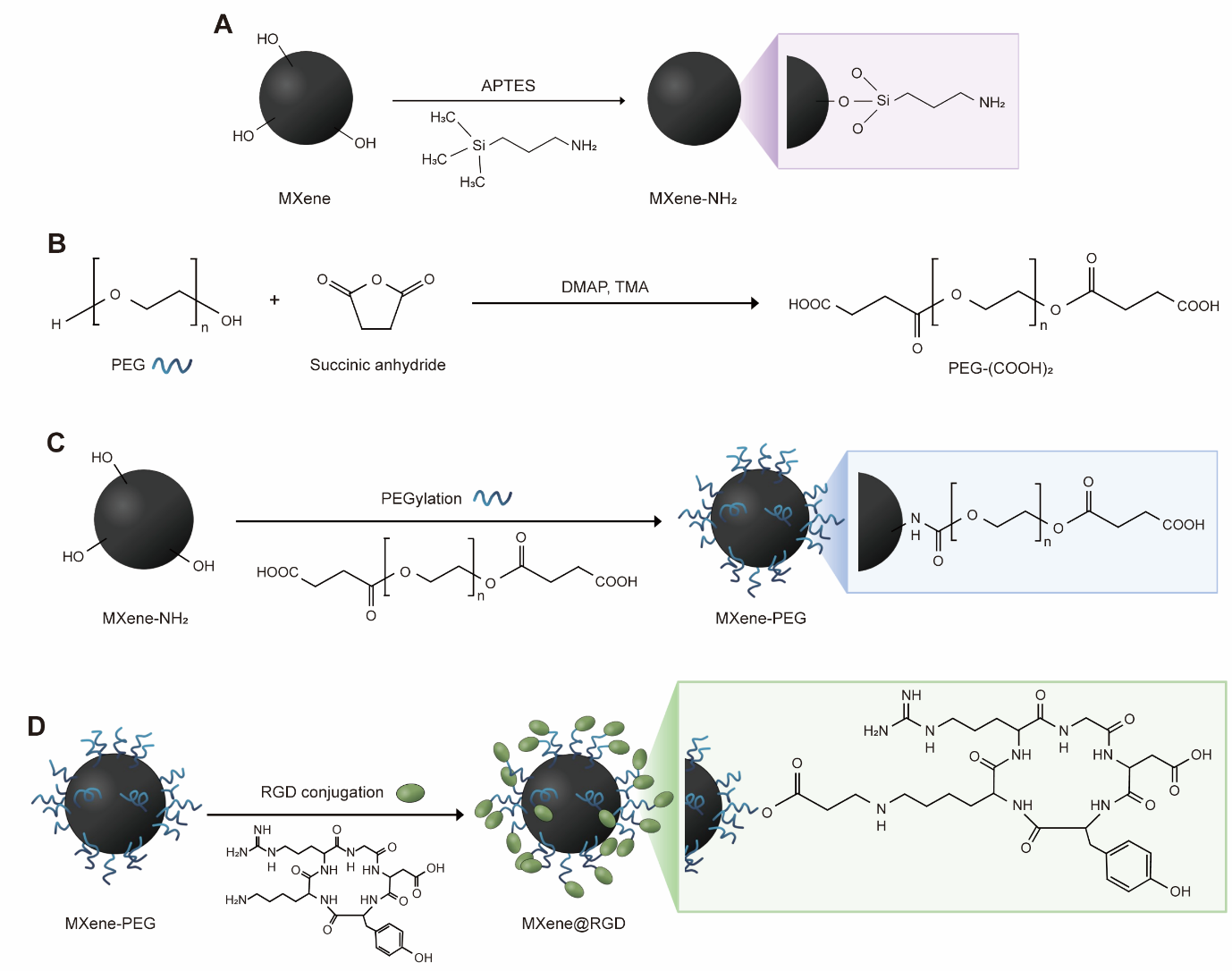


**Figure S5.** Schematic diagrams of the conjugation of PEG and RGD peptide on the surface of MXene particles. (A) A schematic diagram of the amination of MXene. (B) A schematic diagram of the carboxylation of polyethylene glycol (PEG). (C) A schematic diagram of MXene's PEGylation. (D) A schematic diagram of MXene for RGD peptide conjugation.


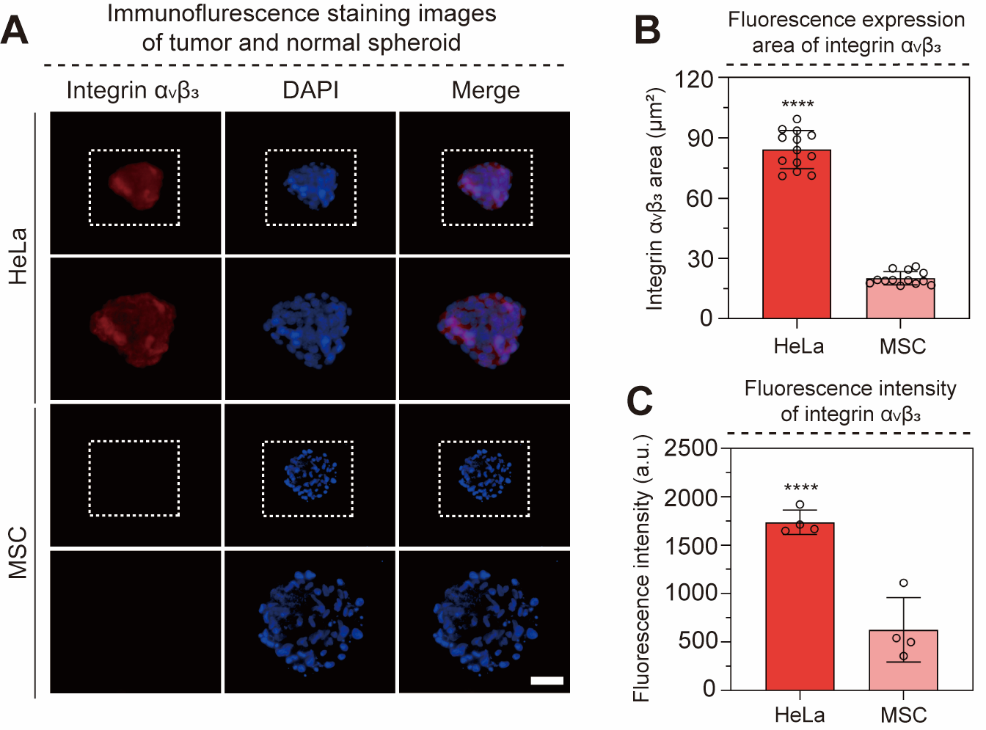


**Figure S6.** Differences in integrin α_v_β_3_ expression between tumor and normal cells for RGD peptides to bound. (A) Immunofluorescence staining image of the tumor and normal cell spheroid (above X400, below X630); red: Integrin α_V_β_3_, blue: DAPI. (B) Fluorescence expression area of integrin α_v_β_3._ (C) Fluorescence intensity of integrin α_v_β_3_. Scale bar: (A) 40 μm (above), 60 μm (below). All data represent mean ± SD (n=3). ****p < 0.0001. The symbol * indicates comparisons with the MSC group.

**
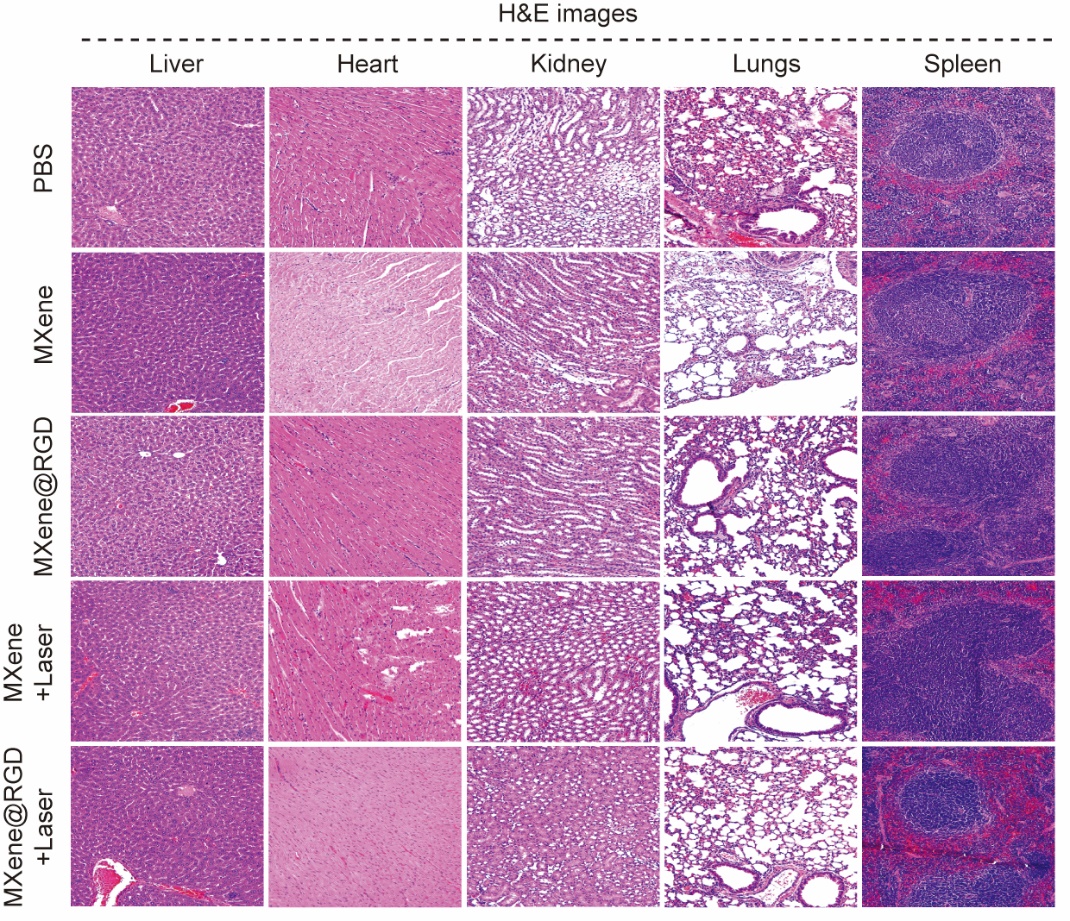
**

**Figure S7.** H&E staining images are used to treat all separated organs, such as the liver, heart, kidney, lung, and spleen, with nanoparticles and then test for tissue damage.
